# Successful Treatment of ALK-Positive Large-Cell Neuroendocrine Carcinoma of the Lung With Sequential ALK Inhibitors: A Case Report

**DOI:** 10.1016/j.jtocrr.2023.100538

**Published:** 2023-06-15

**Authors:** Takayuki Kobayashi, Yuji Uehara, Kageaki Watanabe, Tsunekazu Hishima, Yukio Hosomi

**Affiliations:** a Department of Thoracic Oncology and Respiratory Medicine, Tokyo Metropolitan Cancer and Infectious Diseases Center, Komagome Hospital, Tokyo, Japan; b Department of Pathology, Tokyo Metropolitan Cancer and Infectious Diseases Center, Komagome Hospital, Tokyo, Japan

**Keywords:** ALK fusion, Alectinib, Lorlatinib, LCNEC, Case report

## Abstract

ALK-positive large-cell neuroendocrine carcinoma (LCNEC) is an exceptionally rare form of lung cancer. The efficacy of ALK inhibitors in treating ALK-positive LCNEC remains unclear. Here, we report a case of ALK-positive LCNEC of the lung, which revealed a sustained clinical benefit (24+ mo of overall survival) after treatment with sequential ALK inhibitors and local therapies. This remarkable improvement in survival underscores the importance of testing metastatic LCNEC for biomarkers, such as *ALK* rearrangement, using immunohistochemistry or next-generation sequencing, especially in younger patients.

## Introduction

Large-cell neuroendocrine carcinomas (LCNECs) are a rare subset of lung cancers, accounting for approximately 2% of cases. They exhibit a high-grade neoplasm phenotype and have limited therapeutic options, which results in poor survival outcomes. *ALK* rearrangements are present in approximately 3% to 7% of NSCLC cases, primarily adenocarcinomas,^1^ and are even rarer in LCNEC, with there being only a few reported cases in the literature. Alectinib, a highly selective ALK tyrosine kinase inhibitor (TKI), is the preferred first-line treatment for patients with advanced ALK-positive NSCLC. The ALEX study reported outstanding therapeutic efficacy with a median progression-free survival (PFS) of 34.8 months.^2^ Other ALK inhibitors, such as lorlatinib and brigatinib, also revealed a remarkable survival benefit.^3^^,^^4^ However, the efficacy of ALK inhibitors in treating patients with ALK-positive LCNEC remains uncertain. Here, we report a patient with ALK-positive LCNEC of the lung who achieved prolonged survival after treatment with sequential ALK TKIs and local therapies.

## Case Presentation

**Figure 1:**
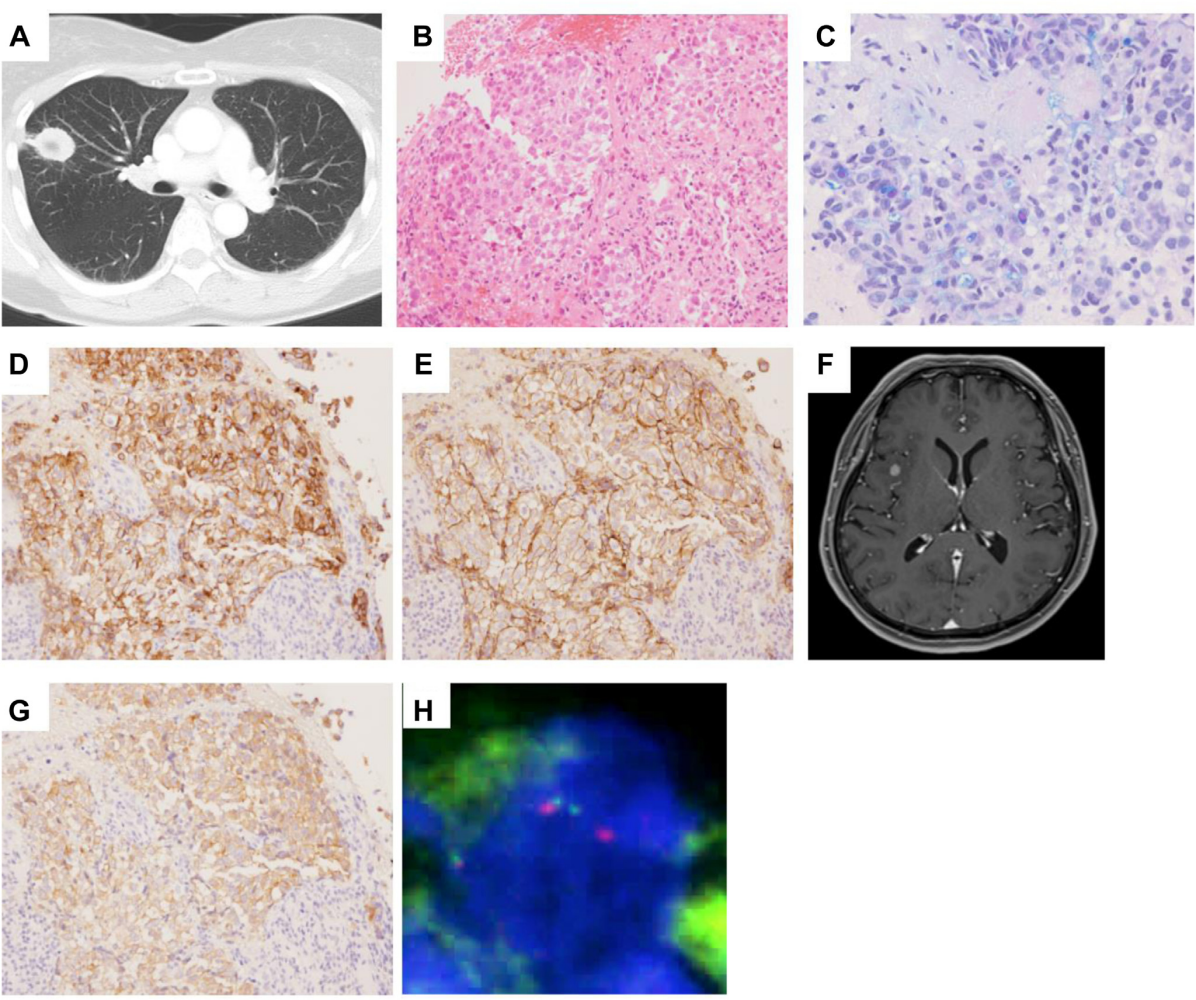
Chest CT at diagnosis (*A*). Histologic characteristics of large-cell neuroendocrine carcinoma from the initial biopsy of the lung tumor (*B*–*E*). Head magnetic resonance imaging at 9 months after chemoradiotherapy (*F*). Histologic characteristics of ALK in the initial biopsy of the lung tumor (*G*, *H*). (*A*) Chest CT illustrating a 2.7 cm right upper lobe nodule. (*B*) HE staining illustrating neuroendocrine structure (organoid nesting) and cytologic features of non–small cell carcinoma (large-cell size, granular chromatin, and prominent nucleoli). (*C*) Alcian Blue and periodic acid-Schiff staining illustrating intracytoplasmic mucin in scattered tumor cells suggestive of glandular differentiation. Immunohistochemical staining exhibiting diffuse immunoreactivity for (*D*) synaptophysin and (*E*) CD56. (*F*) Head MRI illustrating multiple, new brain metastases 9 months after chemoradiotherapy. (*G*) Immunohistochemical staining highlighting diffuse positivity for ALK. (*H*) ALK FISH break-apart probes revealing aberrant staining patterns (one fused and one split red and green). CT, computed tomography; FISH, fluorescence in situ hybridization; HE, hematoxylin and eosin; MRI, magnetic resonance imaging.

**Figure 2:**
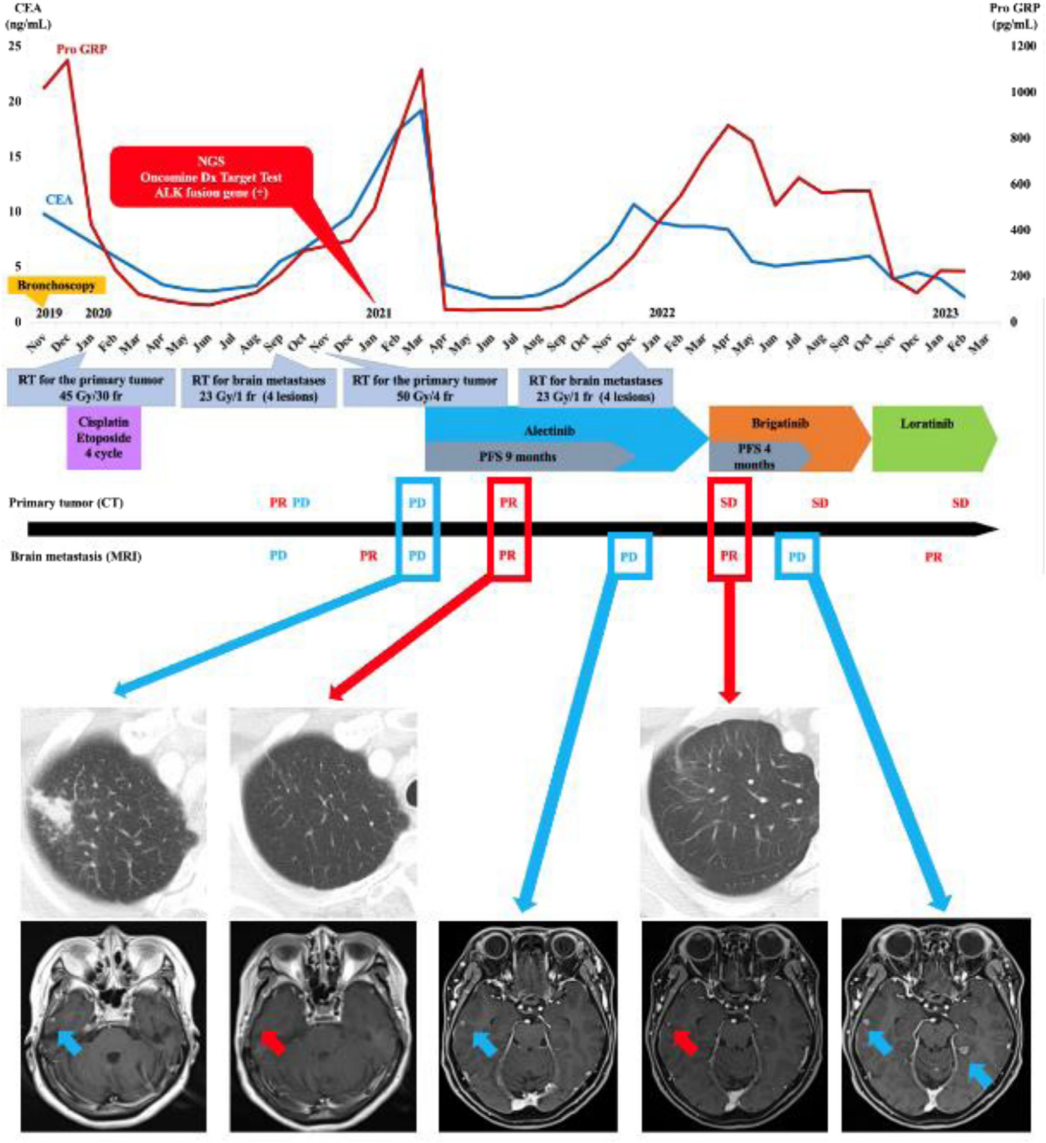
Timeline of the patient’s clinical course. CEA, carcinoembryonic antigen; fr, fraction; Gy, gray; NGS, next-generation sequencing; PD, progressive disease; PFS, progression-free survival; PR, partial response; Pro-GRP, pro-gastrin-releasing peptide; RT, radiotherapy; SD, stable disease.

The patient was a healthy, 57-year-old, Asian woman and former smoker who presented with a productive cough in November 2019. Chest computed tomography revealed opacification in the right upper lobe and adenopathy in the right hilar and paratracheal regions (Fig. 1*A*). Her levels of carcinoembryonic antigen and pro-gastrin–releasing peptide were elevated. Magnetic resonance imaging (MRI) of the head and a whole-body fluorodeoxyglucose-positron emission tomography scan confirmed the absence of metastatic lesions. Bronchoscopy and biopsy revealed a tumor composed of large cells of an organoid nesting pattern (Fig. 1*B*). A small number of tumor cells contained intracytoplasmic mucin suggesting focal glandular differentiation (Fig. 1*C*). Immunohistochemically, the tumor cells stained diffusely positive for synaptophysin (Fig. 1*D*), CD56 (Fig. 1*E*), chromogranin, and TTF-1. These findings were consistent with combined LCNEC and adenocarcinoma. The patient was diagnosed with cT2aN2M0, clinical Stage IIIA (eighth edition TNM), and was treated with concurrent chemoradiotherapy with four cycles of cisplatin and etoposide. The primary lung tumor revealed a partial response (PR) and a decline in tumor markers. Nine months after concurrent chemoradiotherapy initiation, a brain MRI revealed multiple new brain metastases (Fig. 1*F*), and stereotactic radiosurgery was performed. Oligoprogression of the primary tumor was detected 1 month later, and the patient received stereotactic body radiotherapy for the primary tumor with 50 Gy in four fractions; however, four months later, computed tomography scans revealed progression in the primary tumor, brain, and liver. The Oncomine Dx Target Test, a type of next-generation sequencing (NGS), revealed the presence of *ALK* rearrangement, but no other genomic alterations were found. The presence of the *ALK* rearrangement was confirmed by immunohistochemistry (IHC) and fluorescence in situ hybridization analysis (Fig. 1*G* and *H*). First-line treatment with alectinib was initiated in March 2021 and revealed a PR with a decrease in the lung, brain, and liver metastases 4 months later. However, 9 months after alectinib initiation, a repeat brain MRI revealed an increase in multiple brain metastases, leading to the administration of additional stereotactic radiosurgery treatment for the brain. On the detection of multifocal brain disease progression 3 months later, alectinib was switched to brigatinib, resulting in improvement of the extensive brain metastases after 1 month. However, 4 months into brigatinib treatment, multiple new brain metastases were observed, prompting a switch to lorlatinib therapy. After 2 months, a brain MRI revealed shrinkage of the brain metastases, and the treatment is ongoing (24+ mo of overall survival after the onset of ALK TKI therapy). Figure 2 illustrates the timeline of the patient’s treatment.

## Discussion

To the best of our knowledge, only a few patients with ALK-positive LCNEC treated with alectinib have been previously reported (Table 1). This subset of patients typically presents at a younger age regardless of smoking status, which is consistent with our case.^5^, ^6^, ^7^, ^8^, ^9^ Previous studies have reported mixed findings on the efficacy of alectinib in treating ALK-positive LCNEC. Our patient achieved PR after receiving alectinib; however, 9 months of PFS is shorter than typically observed in patients with adenocarcinoma. Although most previous patients achieved PR, PFS associated with alectinib therapy (4–11 mo) seemed shorter than observed in the ALEX study.^2^ The difference in pathologic features could potentially explain the discrepancy in the clinical outcomes. Although some retrospective studies have reported the benefit of alectinib in a tumor-agnostic context, its therapeutic efficacy might vary depending on the tumor type.^10^ Two phase 2 trials (ALpha-T trial and TAPISTRY trial) are currently underway to assess the efficacy of alectinib in patients with ALK-positive tumors other than NSCLC. Further research is needed to determine the efficacy of alectinib in a tumor-agnostic setting.

**Table 1:** Characteristics of ALK-Positive LCNEC Cases

Case	Reference	Age (y)	Sex	Smoking Status	ALK TKI	PFS	Clinical Outcome	OS^a^
1	Tashiro et al. 2020^5^	32	F	Former	Alectinib	11M	PR	11M+
2	Masuda et al. 2021^9^	72	M	Never	Alectinib	4M	SD	4M+
3	Leblanc et al. 2021^7^	37	M	Never	Alectinib Brigatinib Lorlatinib	4M 2W 4W	SD PD PD	8M
4, 5	Akhoundova et al. 2022^6^	37	M	Never	Alectinib Lorlatinib	10M 12M+	PR SD	22M+
		68	F	Never	Alectinib	a few days	PD	a few days
6	Wiedemann et al. 2022^8^	47	F	Former	Crizotinib Alectinib Ceritinib Brigatinib Lorlatinib	3M 10M 4W 4W 8M	SD PR PD PD SD	36M
7	Kobayashi et al. 2023 (our case)	57	F	Former	Alectinib Brigatinib Lorlatinib	9M 4M 3M+	PR SD SD	24M+

ALK TKI, anaplastic lymphoma kinase tyrosine kinase inhibitor; F, female; LCNEC, large-cell neuroendocrine carcinoma; M, male; M, months; NA, not available; OS, overall survival; PD, progressive disease; PFS, progression-free survival; PR, partial response; SD, stable disease; W, weeks.

a After the onset of ALK TKI therapy

In our case, local therapies for oligoprogression and sequential ALK inhibitors prolonged the patient’s survival. At 24 months after the onset of ALK TKI therapy, the patient currently remains in treatment. ALK inhibitor therapy often leads to acquired resistance and disease progression. According to the European Society for Medical Oncology guidelines, when a lesion exhbits progression despite alectinib therapy, local treatments for oligoprogression and switching to another ALK TKI for systemic progression are recommended.^11^ Brigatinib and Lorlatinib have exhibited clinical benefit in patients previously treated with second-generation TKIs with response rates of 34% to 40%.^12^, ^13^, ^14^ We have illustrated the importance of combining local therapies and sequential ALK TKIs in ALK-positive LCNEC.

LCNEC is classified into two distinct subsets with genomic signatures, SCLC and NSCLC.^15^ The former is characterized by *TP53*/*RB1* coalterations and a complete absence of *KRAS*/*STK11*/*KEAP1* mutations whereas the latter is characterized by the absence of *TP53*/*RB1* coalterations and the presence of *KRAS*/*STK11*/*KEAP1* mutations. Although the targeted NGS in our case had limitations, given the *ALK* rearrangement and high TTF-1 expression, the present case might be an instance of the NSCLC-like subset. In daily clinical practice, LCNEC with high TTF-1 or napsin-A expression by means of IHC indicates the NSCLC-like subset, and conducting NGS is worthwhile, as this subset might harbor targetable genomic alterations.

## Conclusions

Sequential ALK TKIs and local therapies led to prolonged survival in a patient with ALK-positive LCNEC. The remarkable improvement in survival seen in the patient underscores the importance of testing metastatic LCNEC for biomarkers, such as *ALK* rearrangement, using IHC or NGS, particularly in younger patients.

## CRediT Authorship Contribution Statement

**Takayuki Kobayashi:** Writing of the first draft, Investigation, Visualization, Software.

**Yuji Uehara:** Conceptualization, Writing of the first draft, Investigation, Supervision.

**Kageaki Watanabe:** Conceptualization, Resources, Investigation, Manuscript review and editing, Supervision.

**Tsunekazu Hishima, Yukio Hosomi:** Investigation, Manuscript review and editing.

**Yukio Hosomi:** Investigation, Manuscript review and editing, Project administration.
